# Optimization of cationic nanoparticles stabilized by poloxamer 188: A potential approach for improving the biological activity of *Aloeperryi*

**DOI:** 10.1016/j.heliyon.2023.e22691

**Published:** 2023-11-21

**Authors:** Tahany Saleh Aldayel, Mohamed M. Badran, Abdullah H. Alomrani, Nora A. AlFaris, Jozaa Z. Altamimi, Ali S. Alqahtani, Fahd A. Nasr, Safina Ghaffar, Raha Orfali

**Affiliations:** a Clinical Nutrition, Department of Health Sciences, Faculty of Health and Rehabilitation Sciences, Princess Nourah bint Abdulrahman University, P.O. Box 84428, Riyadh, 11671, Saudi Arabia; b Department of Pharmaceutics, College of Pharmacy, King Saud University, Riyadh, 11495, Saudi Arabia; c Nanobiotechnology Unit, College of Pharmacy, King Saud University, Riyadh, 11495, Saudi Arabia; d Department of Physical Sports Sciences, College of Sports Sciences & Physical Activity, Princess Nourah bint Abdulrahman University, P.O. Box 84428, Riyadh, 11671, Saudi Arabia; e Department of Pharmacognosy, College of Pharmacy, King Saud University, Riyadh, 11451, Saudi Arabia; f Biology Department, College of Science, Imam Mohammad Ibn Saud Islamic University (IMSIU), Riyadh, 11623, Saudi Arabia

**Keywords:** *Aloe perryi*, CS, SLNs, Optimization, Evaluation, Antioxidant, Antimicrobial, Anticancer, Nanoparticles

## Abstract

Aloe perryi (AP) has gained considerable interest as a medicinal herb in various biological applications due to its rich phytochemical composition. However, the therapeutic benefits of AP could be potentiated by utilizing nanotechnology. Moreover, cationic solid lipid nanoparticles (CSLNs) possess remarkable characteristics that can greatly enrich a variety of biological uses. An optimization approach was used to achieve high-quality CSLNs to maximize the therapeutic efficacy of AP. Therefore, a factorial design was used to investigate the influence of various variables on the attributes of CSLNs quality. In this study, the factors under investigation were compritol 888 ATO (C-888, X1), poloxamer 188 (PL188, X2), and chitosan (CS, X3), which served as independent variables. The parameters measured as dependent variables included particle size (Y1), zeta potential (Y2), and encapsulation efficiency EE (Y3). The relationship among these variables was determined by Analysis of Variance (ANOVA) and response surface plots. The results revealed that PL188 played a significant role in reducing the particle size of CSLNS (ranging from 207 to 261 nm with 1 % PL188 to 167–229 nm with 3 % PL188). Conversely, an increase in the concentration of CS led to a rise in the particle size. The magnitude of positive zeta potential values was dependent on the increased concentration of CS. Moreover, the higher amounts of C-888 and PL188 improved the EE% of the CSLNs from 42 % to 86 %. Furthermore, a concentration-dependent antioxidant effect of the optimized AP-CSLNs was observed. The antioxidant activity of the optimized AP-CSLNs at 100 μg/mL was 75 % compared to 62 % and 60 % for AP-SLNs and AP solution, respectively. A similar pattern of improvement was also observed with antimicrobial, and anticancer activities of the optimized AP-CSLNs. These findings demonstrated the potential of AP-CSLNs as a carrier system, enhancing the biological activities of AP, opening new possibilities in herbal medicines.

## 1 Introduction

Aloe perryi (AP) is a medicinal herb known for its biological properties making it as a promising candidate for various therapeutic applications [[1], [2], [3]]. AP displays different pharmacological activities including antioxidant, antimicrobial, anti-inflammatory, and anticancer activities [[1], [2], [3], [4]]. Additionally, it has been used in various skincare products for centuries [3]. The therapeutic attributes of AP can be ascribed to its rich of essential components, including polysaccharides, vitamins, enzymes, and flavonoid compounds [5,6]. Moreover, AP extract comprises phenolic compounds such as anthrones and proanthocyanidins, showing considerable potential for several biological activities [2]. However, the clinical application of AP is often hindered by poor solubility, low stability, limited permeability, and insufficient therapeutic efficacy [2,7].

There has been a growing interest in the development of delivery systems for herbal drugs, which are designed to boost their therapeutic efficacy [8]. Furthermore, the encapsulation of herbal drugs in a carrier system could protect them from degradation, oxidation, and enzymatic degradation, thereby preserving their bioactivity [9]. Therefore, solid lipid nanoparticles (SLNs) have emerged as promising carriers for the delivery of herbal extract due to their unique properties such as controlled drug release, improved stability, and enhanced bioavailability [10]. Thus, the loading of herbal extracts into SLNs may provide an effective solution to augment their activity [5]. Compritol 888 ATO (C-888) is considered one of the most used materials in SLNs formulation, attributed to its distinctive physicochemical properties [11]. Furthermore, poloxamer 188 (PL188) is an amphiphilic polymer that is widely investigated for improving the stability and physical properties of SLNs [12]. For instance, the stability of SLNs in the presence of PL 188 was improved which was attributed to the protection layer (steric barrier) that PL 188 creates around the particles [13]. The amphiphilic characteristic of PL188 may increase the interaction of SLNs with both the hydrophilic and hydrophobic components. Therefore, the presence of PL188 on the SLN could facilitate the incorporation of a higher amount of the herbal extract into the lipid matrix [13]. PL188 is preferred over Tween 80® as a surfactant in the preparation of SLNs due to its excellent emulsifying and stabilizing properties [12]. PL188 is known as a safer choice for biomedical applications due to its low toxicity profile. PL188 can modulate drug release rates, providing controlled and sustained release profiles, which are often desirable for optimizing drug therapy [13]. Therefore, the use of PL188 as a surfactant in the preparation of SLNs can ameliorate the therapeutic efficacy of loaded agents like AP extract.

The increased efficacy of SLNs has been obtained by their surface modification with biocompatible polymers. Thus, the cationic agents on the surface of SLNs could enhance the activity of herbal extract [5]. The high electrostatic interaction between the positively charged SLNs and the negatively charged cell membrane was expected and facilitated the cellular uptake and intracellular delivery of the herbal medicine [[5], [6], [7], [8], [9], [10], [11], [12], [13], [14]]. Therefore, chitosan (CS)-surface modified SLNs (CSLNs) could be considered as a promising cationic carrier for the significant impact on the delivery of AP [[5], [6], [7], [8], [9], [10], [11], [12], [13], [14], [15]]. The presence of amino groups in CS structure imparted the cationic properties of the nanocarriers. CS is a biodegradable, biocompatible, and mucoadhesive polymer e that gained significant attention in drug delivery systems. Hence, this study hypothesizes that the CSLNs offer considerable promise for enhancing the therapeutic outcomes of AP such as antioxidant, antibacterial, and anticancer activities.

Therefore, the optimization process is an essential tool to obtain high-quality CSLNs based on their particle size, size distribution, zeta potential, and encapsulation efficiency (EE) to magnify the therapeutic efficiency of AP [16,17]. In addition, it is preferred to use PL188 instead of tween 80® due to its high magnitude. Therefore, this study aims to optimize the formulation parameters of AP-loaded CSLNs, including C-888, PL188, and CS concentrations to achieve the desired physicochemical properties. The optimization process requires a systematic approach, including experimental design methodology such as factorial design to evaluate different parameters and their interactions to identify the optimal composition.

## 2 Material and methods

### 2.1 Materials

Compritol 888 ATO (C-888) was obtained from Gattefosse, Chemin de Genas (Saint-Priest, France). Phospholipid (PL, Lipoid S100, purity >98 % of phosphatidylcholine) was purchased from Lipoid GmBH (Ludwigshafen, Germany). Low molecular weight chitosan with a 96 % degree of deacetylation and poloxamer 188 were both obtained from Sigma-Aldrich Chemie, GmbH (Steinheim, Germany). All reagents and chemicals used for chromatography were of ultra-performance liquid chromatography grade. All other chemicals and organic solvents were of reagent grade.

### 2.2 AP collection and extraction

The dried resin portions of AP Baker, commonly known as Saber Socotri, were acquired from the local market in Sanaa, Yemen. The dried AP was then crushed into a powder and carefully kept in an airtight bottle. To prepare the AP extract, 50 g of the dried-powder was subjected to maceration with 100 mL of absolute methanol for 48 h and then sonicated for 30 min at 25 °C. This extraction procedure was reiterated twice, using 100 mL of methanol on each time. The supernatant obtained from each extraction was gathered and concentrated using a rotary evaporator (Model 011) from Buchi, Flawil, Switzerland) set at 60 °C. For the spectrophotometric assay, the dried extract was dissolved in methanol and then passed through a syringe filter (0.22 Pa TFE) (Fisher Scientific, Pittsburgh, PA, USA) to ensure purity and remove any particulate matter. The yield value of the obtained AP was detected with the following equation:

(1) $$\text{Yield}\left(\%\right)=\frac{W1}{W2}\times 100$$

where W1 represents the extract weight after the evaporation and W2 is AP-dried weight.

### 2.3 Oprimization

To optimize AP-loaded CSLNs, three factors, three levels (3^2^) as a full factorial design was investigated by a statistical package (Design-Expert 11). A randomized Box-Behnken experimental design was used to characterize the effect of three independent factors, namely lipid concentration (X1), PL188 concentration (X2), and CS concentration (X3), on the qualities of AP-loaded CSLNs (Table 1). Statistical models including main, quadratic, and interactive modes were obtained to evaluate the effect of the independent factors on particle size (nm; Y1), zeta potential (mV; Y2), and EE (%; Y3).

**Table 1:** Variables in the Box-Behnken factorial design for the AP-loaded CSLNs.

Independent Variable, factor	Low (−1)	Medium (0)	High (1)
X1: C-888 (%)	2	3	4
X2: PL188 (%)	1	2	3
X3: CS (mg/mL)	0.5	1	2
Dependent variable, response			
Y1: Particle size (nm)			
Y2: Zeta potential (mV) Y3: Encapsulation efficiency (EE%)			

### 2.4 Preparation of AP-loaded CSLNs

The composition of the AP-loaded CSLNs from F1 to F15, provided from the experimental factorial design, is illustrated in Table 2. Firstly, the AP-SLNs were made with the solid lipid (C-888) and PL 188 using the ultrasonic melt-emulsification technique [18]. Lipoid S100 was added to all formulations at a constant amount (1 %). Briefly, a clear molten solution was produced by heating the lipid phase to 85 °C. The melted lipid was then mixed with the hot PL 188 aqueous phase (85 °C) using an Ultra Turrax T25 (Janke and Kunkel, Staufen, Germany) at 8000 rpm for 10 min. The obtained dispersion was probe-sonicated for 5 min at 60 % amplitude using a Bandelin Sonopuls HD 220 (Bandelin Electronics, Germany). To eliminate any aggregated and titanium particles resulting from probe sonication, light centrifugation was employed at 3000 rpm for 3 min.

**Table 2:** Composition and properties of AP-loaded CSLNs.

Run	C-888 (%)	PL188 (%)	CS (mg/mL)	X1: Particle size (nm)	X2: Zeta potential (mV)	X3: EE (%)
F1	2	1	0.5	207.3 ± 8.1	18.5 ± 1.2	57.6 ± 6.1
F2	4	3	1.5	228.7 ± 6.8	25.1 ± 1.8	76.7 ± 8.2
F3	4	3	0.5	185.6 ± 6.2	16.4 ± 0.9	84.6 ± 9.0
F4	3	2	1.5	217.3 ± 8.5	32.3 ± 2.2	62.9 ± 6.6
F5	4	1	1.5	243.1 ± 11.0	30.3 ± 2.1	80.6 ± 7.8
F6	3	2	0.5	176.6 ± 6.1	13.4 ± 0.9	73.7 ± 7.3
F7	2	2	1	196.2 ± 5.9	25.5 ± 1.7	42.3 ± 4.5
F8	3	3	1	193.3 ± 4.8	21.1 ± 1.5	84.6 ± 5.1
F9	2	3	1.5	223.7 ± 6.7	31.4 ± 2.2	67.8 ± 4.9
F10	2	1	1.5	261.4 ± 5.4	31.8 ± 2.1	61.9 ± 3.2
F11	3	2	1	152.5 ± 9.3	27.6 ± 1.9	82.6 ± 7.2
F12	3	1	1	254.6 ± 9.5	26.2 ± 1.8	68.8 ± 5.7
F13	4	1	0.5	238.8 ± 2.1	17.3 ± 1.3	81.6 ± 11.3
F14	4	2	1	236.3 ± 8.3	23.9 ± 1.6	86.5 ± 7.1
F15	2	3	0.5	166.9 ± 11.5	17.3 ± 1.2	55.1 ± 2.8

To prepare the CSLNs, the physical adsorption method was applied [19]. Accurate weight of CS was dissolved in 0.2 % v/v of acetic acid to get a solution of CS (1 % w/v in). This solution was filtered through 0.8 mm to remove any clumps. Then, the CS solution was gradually introduced into the dispersion of SLNs with an equivalent volume under stirring at a room for 4 h to form CSLNs. Moreover, blank CSLNs were produced in the same manner without AP extract.

### 2.5 Characterization of AP-CSLNs

#### 2.5.1 UV–visible spectrophotometry

The maximum wavelengths of AP extract, plain CSLNs, and AP-CSLNs absorption were identified. They were diluted serially at different concentrations (2–10 μg/mL). Using a UV–visible spectrophotometer (Thermo Scientific GENESYS 10S UV-VIS, Madison, USA), the absorbance of these samples was then detected, and a calibration curve was created to prove the linearity.

#### 2.5.2 Particle size and zeta potential investigation

A Zetasizer Nano ZS (Malvern Instruments, Worcestershire, UK) was employed for assessing particle size and polydispersity index (PDI) based on dynamic light scattering mode. Furthermore, zeta potential was measured through electrophoretic light scattering mode. The obtained CSLNS were diluted at a ratio of 1–500 with deionized water, transferred to a quartz cuvette, and inspected at a scattering angle of 90°. All measurements were done in triplicate at room temperature.

#### 2.5.3 Encapsulation efficiency (EE) and stability studies

The prepared formulations were first subjected to light-centrifugation at 3000 rpm for 3 min to remove undissolved AP. The encapsulated amount of AP was determined using an indirect method [20]. The total amount of AP (AP_Total_) was determined using a UV–vis spectrophotometer (Thermo Scientific GENESYS 10S UV-VIS, Madison, USA) at a wavelength of 297 nm after proper dilution with methanol. The supernatant (AP_Free_) was aspirated after centrifugation of AP formulation at 40000 rpm for 30 min (Beckman Coulter, Pasadena, CA, USA). The supernatant (AP_Free_) was quantified by a UV–vis spectrophotometer at 297 nm. All measurements were quantified three times. The following equation was used to ascertain EE (%).

(2) $$\text{EE}\left(\%\right)=\frac{{\text{AP}}_{\text{Total}}‐{\text{AP}}_{\text{Free}}}{{\text{AP}}_{\text{Total}}}x100$$

Moreover, the stability study was conducted for the optimized AP-CSLNs formula by measuring particle size, zeta potential, and EE% after storage at 4 °C for one month.

#### 2.5.4 *In vitro* release study

The release profiles of the optimized AP-loaded CSLNs, AP-SLNs, and AP extract (ethanolic solution) were examined. A certain volume of the obtained samples, corresponding to 1 mg of AP, was loaded into dialysis bags with a molecular weight cut-off of 12–14 kDa and then sealed. These bags were then kept in a container holding 20 mL of receptor fluid (PBS pH 7.4) containing 0.2 % w/v Tween80 under stirring of 100 rpm and temperature of 37 °C using a water bath. Samples from receptor media were taken at predetermined intervals up to 48 h and replenished with an equal volume of fresh buffer to preserve sink conditions. The collected samples were centrifugated at 10000 rpm for 5 min and the amount of AP in the resulting supernatant was quantified by a UV spectrophotometer (Thermo Scientific GENESYS 10S UV-VIS, Madison, USA) at 297 nm. The experiment was performed three times. The release kinetics of AP were established by fitting the data to various release kinetic models (zero-order, first-order, Higuchi, and Korsmeyer-Peppas).

Moreover, the morphology of optimized AP-CSLNs was observed using the transmission electron microscope (TEM) at a voltage of 60 kV (JEM1011, JEOL, Tokyo, Japan). The sample was appropriately diluted and negatively stained using a 1 % (w/v) uranyl acetate solution on a copper grid. After removing the excess liquid from the grid with filter paper and air-drying, the samples were visualized using the TEM.

#### 2.5.5 Antioxidant activity

The evaluation of the total antioxidant activity of AP extract, AP-SLNs, and optimized AP-CSLNs was based on their capability to scavenge 2,2-diphenyl-1-picrilhidrazil (DPPH) as reported previously [21,22]. A defined volume (500 μL) of various concentrations of AP extract, AP-SLNs, and optimized AP-CSLNs was mixed with 125 μL of a DPPH (0.02 %) methanolic solution and incubated for 1 h in the dark at room temperature.

Antioxidant activity was indicated by a color shift from violet to colorless. A DPPH and ascorbic acid solutions were used as a blank and positive controls. After incubation, the all samples were measured spectrophotometrically at 517 nm against a blank sample. The experiments were conducted in triplicate. and the subsequent equation was applied to determine the activity of free radical scavenging:

(3) $$\text{Free}\;\text{radical}\;\text{scavenging}\;\text{activity}\;\left(\%\right)=\frac{{\text{Abs}}_{\text{blank}}{‐\text{Abs}}_{\text{sample}}}{{\text{Abs}}_{\text{blank}}}x100$$

#### 2.5.6 Antimicrobial activity

The antimicrobial efficacy was appraised using the microdilution broth with minor modifications in accordance with the established protocols of the Clinical and Laboratory Standards Institute (CLSI, 2012) [23]. The reference strains of *Staphylococcus aureus* (*S. aureus*) ATCC 29213 and *Pseudomonas aeruginosa (P. aeruginosa)* ATCC 27853 were used. They were obtained from the American Type Culture Collection (Rockville, MD, USA). The obtained formulations were diluted to achieve 200 μg/mL of AP, which were subsequently introduced into 96-well plates. To prepare the bacterial suspension, the colonies with similar morphology obtained from 24 h old culture were introduced into PBS. This suspension was then diluted to 0.5 McFarland standard with an optical density ranging from 0.08 to 0.1 at a wavelength of 625 nm. To achieve 5 × 10^5^ CFU/mL, the bacterial suspension was further diluted at a ratio of 1:100 in media and then 50 μL was dispersed onto each plate. The plates were placed in an incubator for 24 h at 37 °C. The bacterial growth was evaluated by observing the color change from yellow to red after the addition of 0.2 mg/mL *p*-Iodonitrotetrazolium. Furthermore, the minimum inhibitory concentration (MIC) was also determined by two-fold serial dilutions from stock sample solutions in Muller Hinton broth (MHB). Afterward, 100 μL of these dilutions were added to 96-well plates, creating a concentration range from 100 to 0.75 μg/mL. Bacterial strains were cultivated aerobically at 37 °C on Mueller-Hinton agar. Reference drug such as penicillin was used, and DMSO served as a positive control. MIC values were visually noticed by shifting the color from yellow to red, which was measured spectrophotometrically at 600 nm by μQuant (BioTek Instruments, Winooski, VT, USA). The reading of control plates (without bacteria) was used as background absorbance. Each assay was repeated three times independently. Samples exhibiting MIC values ≤ 100 μg/mL were indicated as markedly potent [24].

#### 2.5.7 Cytotoxicity activity

The effect of AP extract, AP-SLNs, and the optimized AP-CSLNs on the viability of different human cancer cell lines was conducted using an MTT assay. Doxorubicin was used as a positive control. The cell viability of lung carcinoma cells (A549), colon cancer cells (LoVo), and breast cancer cells (MCF-7) was studied. The cells were grown in Dulbecco's Modified Eagle Medium (Gibco, Grand Island, NY, USA), supplemented with 10 % fetal bovine serum and 10 % penicillin-streptomycin. The cultures were preserved at 37 °C in a 5 % CO_2_ incubator. The cells were placed in 96-well plates and exposed to varying concentrations of AP extract, AP-SLNs, and AP-CSLNs for a duration of 48 h. The medium was discarded and the wells were washed. Then, MTT solution was placed in the plates followed by incubation for 4 h. The resulting MTT-formazan crystals were dissolved in acidified isopropanol, and the absorbance was quantified at 570 nm by a microplate reader (BioTek Instruments, Winooski, VT, USA). The following formula was adopted to calculate the cell viability based on the readings of absorbance [25].

(4) $$\text{Cell}\;\text{viability}\;\left(\%\right)=\frac{\text{Absorbance}\;\text{of}\;\text{treated}\;\text{cells}}{\text{Absorbance}\;\text{of}\;\text{untreated}\;\text{cells}}X100$$

### 2.6 Statistical data analysis

The analysis of data was conducted using Microsoft Excel, Version 2010, and Origin software, version 8. The results are presented as mean ± standard error (n = 3). For the comparison of three or more conditions, a one-way analysis of variance (ANOVA) was applied with p < 0.05.

## 3 Results and discussion

### 3.1 The yield production

The yield product of dry AP was 61.4 %. The relatively low amount of AP could be associated with the solvent volume and the purification process [7].

### 3.2 Preparation and characterization of AP-loaded CSLNs

The Box-Behnken factorial design created 15 formulations (Table 2), and the results of the statistical analysis highlighted the significant outcomes as indicated by p < 0.05 (Table 3). Moreover, the analysis in the program covered the individual variables (C-888, PL188, and CS concentrations), their quadratic, and the interactive effects such as PL188-CS.

**Table 3:** Analysis of variance for the effect of lipid (X1), PL188(X2), and CS (X3) on the responses of AP-loaded CSLNs. (The significant level *p* = 0.05).

Responses	Source	Sum of Squares	F-Ratio	*P*-Value
Particle size (nm); Y1	A: C-888	598.04	0.99	0.366
	B: PL188	4298.71	7.09	0.045
	C: CS	3973.38	6.55	0.051
	AB	14.22	0.02	0.884
	AC	501.39	0.83	0.405
	BC	213.56	0.35	0.579
Zeta potential (mV); Y 2	A: Lipid	12.72	1.78	0.240
	B: PL188	16.36	2.28	0.191
	C: CS	463.78	64.74	0.001
	AB	2.67	0.3721	0.569
	AC	4.15	0.5791	0.481
	BC	1.49	0.2086	0.667
EE (%); Y 3	A: Lipid	207.28	1.91	0.013
	B: PL188	1569.50	14.45	0.207
	C: CS	32.69	0.3011	0.941
	AB	0.6616	0.0061	0.889
	AC	2.32	0.0214	0.419
	BC	84.17	0.7751	0.959

The hot homogenization-ultrasonication technique was used for the production of SLNs successfully due to its efficiency, simplicity, and reliability. This method has the advantages of avoiding the organic solvent evaporation step and producing a uniform and homogenous SLNs dispersion. In preliminary work, AP-SLNs were constructed using a variety of lipids, including stearic acid, C-888, and precirol ATO5, to find the most effective lipids for the loading of AP. C-888 was selected as a lipid core for SLNs based on the highest encapsulation of AP. C-888 is one of the most common solid lipids used in a variety of pharmaceutical delivery systems. C-888 is generally recognized as safe (GRAS) and has been widely used in the production of SLNs [11]. To highly stabilize AP-CSLNs, PL188 as a surfactant and lipoid S100 as a cosurfactant (LP) were incorporated in the SLNs formula. The prepared SLNs were then coated with CS to improve their properties. The authors aimed to assess the effect of C-888, PL188, and CS concentrations on AP-loaded CSLNs quality parameters, such as the size of NPs, zeta potential, and EE%. It was found that the CSLNs had excellent features, such as positive charge, biocompatibility, bio-adhesion, and cellular absorption [26].

#### 3.2.1 Particle size and zeta potential investigation

The particle size of SLNs has garnered a lot of consideration as a significant factor in drug delivery. The surfactants are the most investigated excipients used during the production of SLNs for the purpose of increasing drug loading and reducing particle size [27]. They might modify the surface of SLNs, which prevents particle aggregation and creates stable systems [5]. Therefore, selecting an appropriate surfactant is essential for generating effective SLNs with nanometer-scale. PL188 is one of the effective surfactants used to prepare SLNs and improve the drug release profile [17].

Fifteen formulations of AP-CSLNs were evaluated for their particle size (Y1), zeta potential (Y2), and EE% (Y3), (Table 2). The particle size values of the prepared CSLNs were presented in Table 2 and ranged from 152.5 ± 9.3 to 261.4 ± 5.4 nm. The most significant variable affecting particle size (Y1) was PL188 (X2) (*p* = 0.045), followed by CS (X3) (*p* = 0.051), Table 3. The particle size of the prepared AP-CSLNs was not affected by the other independent variables. In addition, it's important to note that all interactive effects did not show statistical significance on the particle size *(p* ≥ 0.05). The effect of the independent variables on the particle size can be ordered, using the sum of the squares, as follows: PL188 (B) > CS (C) > C-888 (A) > AC > BC > AB. In addition, no significant quadratic impact of the independent parameters on the particle sizes was found. The particle size of AP-CSLNs was reduced when PL188 concentration increased from 1 % to 3 %, (Table 2). Accordingly, F10 with 1 % PL188 (2 % C-888, 1 % PL 188, and 1.5 mg/mL CS) has a particle size of 261 nm, whereas F15 with 3 % PL188 (2 % C-888, 3 % PL188, and 0.5 mg/mL CS) showed a particle size of 167 nm.

The particle size of SLNs containing 1 % PL188 was in the following order: F10 > F12 > F5 > F13> F1, and for those containing 2 % PL188 was F14 > F4 > F7 > F6> F11, and with 3 % PL188 as F2 > F9 > F8 > F3 > F15. Moreover, the particle size was in the following order: F13 > F1 > F3 > F6 > F1, using 0.5 mg/mL CS, and similar findings were obtained, applying 1 mg/mL as F12 > F14 > F7 > F8 > F11 and 1.5 mg/mL of CS as F10 > F5 > F2 > F9 > F4. For CS, the increase in the concentration from 0.5 to 1.5 mg/mL using 3 % w/v PL 188 resulted in an increase in the particle size from 167 nm to 223.7 for F15 and F9, respectively, while a non-significant *(p* > 0.05) effect was obtained by increasing CS concentration from 0.5 % to 1 % (w/v). Generally, the particle size of SLNs tends to decrease with increasing PL188 and decreasing CS concentrations. PL188, as a surfactant with a high HLB value (29) [12], has high emulsification properties due to its distinct hydrophilic and lipophilic ends. PL188 can decrease the surface tension, modifying the diffusion of w/o interface of NPs dispersions, which results in a reduction in the particle size of CSLNs [14,28,29]. CS has several benefits, such as high membrane permeability, bio-adhesion properties, and low toxicity [14]. It has been shown that CS nanocarriers increased the biological activity of the loaded herbal extract (Jatropha pelargoniifolia) [30]. The particle size of CSLNs is significantly increased, Table 3, as a result of the presence of CS on the surface of SLNs [5,14]. The PDI values of the obtained formulations were less than 0.5 indicating that the particle size distribution of all formulations is homogeneous.

The optimization of the independent variables during the development of CSLNs is important to achieve the desired zeta potential for the intended application. The impact of each variable and their interactions is critical for tailoring the zeta potential to optimize the stability, AP encapsulating, and targeted delivery of CSLNs [31]. One of the independent variables significantly impacted is the zeta potential of the CSLNs formulations (Table 3). The data presented in Table 3 indicated that the CS (C) had a significant impact *(P* = 0.001) on the zeta potential of the developed CSLNs. In contrast, neither PL188 (*P* = 0.191) nor C-888 (*P* = 0.290) had a considerable influence on the values of the CSLNs' zeta potential. Furthermore, all interactive effects did not have statistical significance on zeta potential (*p* ≥ 0.05). The relationship between zeta potential and CS concentration has been reported in the literature [32]. The obtained CSLNs have positive zeta potential values ranging from 13.4 up to 32.3 mV (Table 2). The particles tended to be more positively charged after coating with CS [14]. The inversion of the zeta potential of the CSLNs was consistent with the successful coating of the SLN's surface with positively charged CS [19,30]. The positive charged NPs have mucoadhesive characterization, which provide interaction with the negatively charged mucin in mucus [32]. This interaction can prolong the residence time cause greater absorption of AP within mucosal surfaces. Therefore, modifying zeta potential by increasing the CS concentration in a formulation can potentially result in a higher positive charge, leading to amended mucoadhesion and particle stability.

#### 3.2.2 Encapsulation efficiency (EE)

**Fig. 1:**
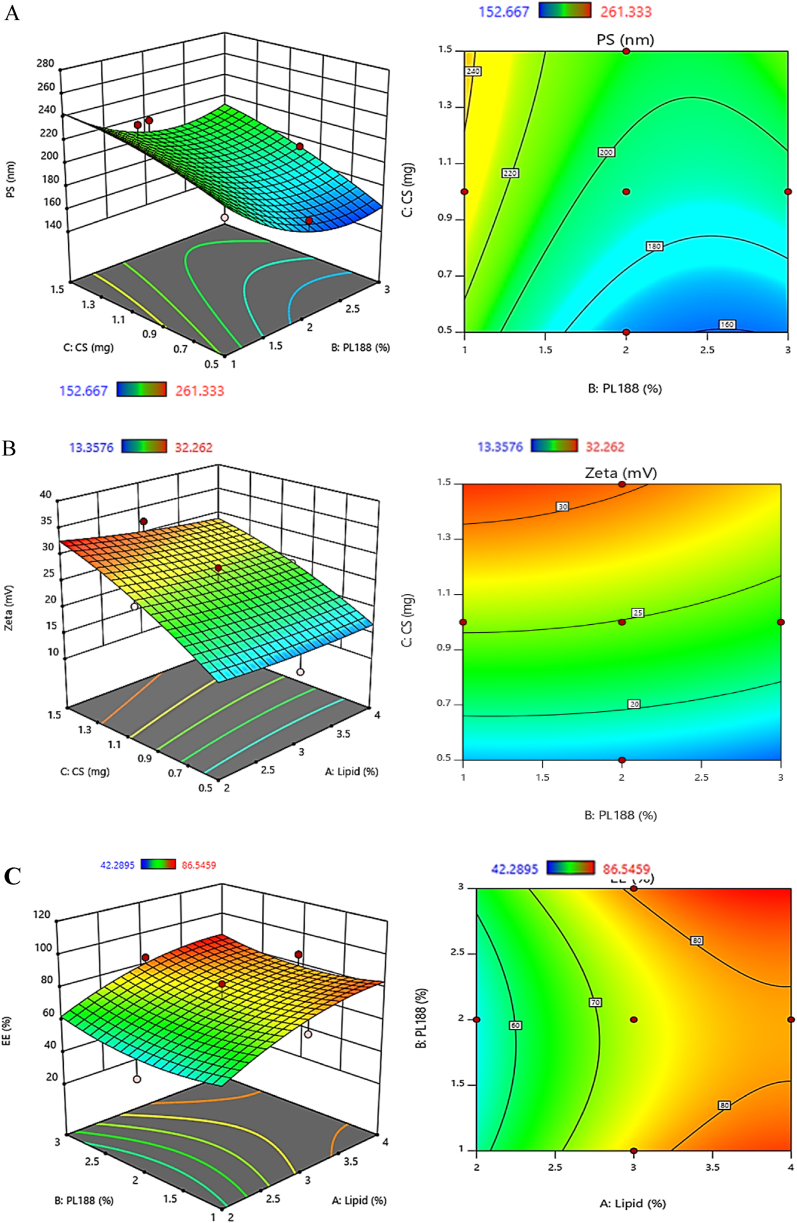
Estimation of response surface plot of the effect of independent factors on AP-CSLNs; particle size (A), zeta potential (B), and EE % (C).

The effect of the independent factors on the EE% of AP-loaded CSLNs is illustrated in Table 2 and Fig. 1C. Analysis of variance (Table 3) revealed that C-888 had a highly significant effect (P = 0.013) on the EE% of AP-loaded CSLNs. Moreover, the quadratic effect of the interaction of C-888 with CS had less effect on the EE% (*P* = 0.419). It was observed that the effect of the concentration of C-888 was significant, whereas PL188 (X2) showed only mild influence (*p* = 0.207). While all interactive effects did not detect statistical significance on EE% (*p* ≥ 0.05). The EE% values for AP-SLNs were in the range of 42.3 ± 4.5 to 86.5 ± 7.11(Table 2). The obtained formulations F3, F5, F8, F11, F13, and F14 showed the highest EE% (>80 %). The prepared CSLNs with a high content of C-888 and PL 188 exhibited high EE% of AP. This effect of C-888 may be attributed to the presence of more space within the SLN structure, which accommodates more of the drug during the preparation. Moreover, C-888 has an imperfection crystalline lattice, resulting in a more disordered structure compared to other solid lipids [11]. This imperfection in the lattice allows for more spaces within the structure resulting in higher drug loading [17]. Moreover, the diffusion rate of the drug into the exterior phase diminishes due to the high viscous lipid phase. This leads to a higher EE%, as demonstrated by previous studies [33]. The reasonable EE% in the present study may be recognized as the proper choice of solid lipid and surfactant. Compared to the previously investigated solid lipids, C-888 is more efficient in terms of drug EE% offering greater space for drug loading because of its less perfection [11]. On the other hand, PL188 (X2) may have a slight impact on the EE% of SLNs due to its surfactant properties and interactions with the lipid matrix [14].

As a surfactant, PL188 can help in stabilizing the SLNs preventing aggregation by dropping the interfacial tension. It has also a positive impact on the solubility of lipophilic drugs within the lipid matrix providing a high drug-loading capacity for SLNs [34]. Moreover, the high concentration of CS (X3) with low amounts of PL188 (X2) and C-888 (X1) demonstrated a negative influence on EE%. This observation can be attributed to the more partitioning phenomenon of CS on the surface of SLNs, which results in a decrease in EE% of AP extract. The response surface plot typically shows contours and three-dimensional surfaces (3D) that illustrate the relationship between the response variables (particle size, zeta potential, and EE %) and the dependent variable (C-888, PL188, and CS concentrations) (Fig. 1). By examining the contours or surfaces formed by the analysis, valuable insights can be obtained regarding the shape and direction of these patterns. This allows for a comprehensive understanding of the individual and combines the independent variables and the dependent variables. A larger CS concentration resulted in an increased particle size value, as seen by the 3D response surface plot, while a higher PL188 concentration resulted in a decreased particle size value (Fig. 1A). The 3D response surface demonstrated the positive zeta potential value as the concentration of CS was increased (Fig. 1B).

#### 3.2.3 Optimization formula

**Fig. 2:**
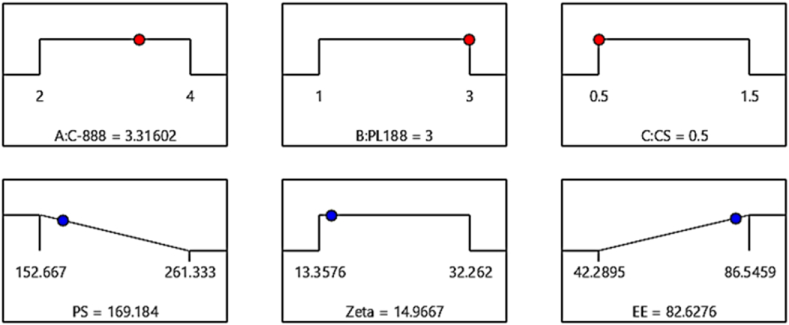
Desirability of the optimized AP loaded CSLNs and the target responses.

The optimized AP-loaded CSLNs formulation was created using the statistical software program (Fig. 2). The recommended optimum conditions for CSLNs formula were 3.3 % C-888, 3 % PL188, and 0.5 % CS. Table 4 provides the predicted and observed values for the optimized AP-CSLNs formula. The values for particle size, zeta potential, and EE% are compared. For particle size, the predicted value was 169.23 nm, while the observed value was 181.4 ± 6.2 nm. This indicates a slight deviation from the predicted value. The predicted value of zeta potential was 14.97 mV, while the observed value was 17.2 ± 3.5 mV. Again, there is a slight difference between the observed values and predicted values. For EE%, the predicted value was 82.63 %, while the observed value was 80.7 ± 4.9 %, which is slightly lower than the predicted value. Overall, the observed values for particle size, zeta potential, and EE% are relatively nearby to the predicted values, indicating a good relationship. However, the slight variation between the predicted and observed values is expected and could be attributed to normal variation between batch to batch production based on the stability results, AP-CSLNs maintained their particle size, zeta potential, and EE values after one month of storage.

**Table 4:** The optimized AP-CSLNs composition, the target responses, and their respective observed and predicted values.

Optimized formula composition	Response	Target	Predicted	Observed
C-888: 3.32 %	Y1: Particle size (nm)	Minimum	169.2	181.4 ± 6.2
PL188: 3 %	Y2: Zeta potential (mV)	Maximum	14.97	17.2 ± 3.5
CS: 0.5 %	Y3: EE (%)	Maximum	82.63	80.7 ± 4.9

Uncoated SLNs were prepared to assess the influence of CS coating on enhancing the desired properties of AP-CSLNs, thus gaining a comprehensive understanding of the overall impact of the coating on the formulation of the nanoparticles. Then, the uncoated optimized AP-SLNs exhibited a particle size of 172.3 ± 2.3 nm, a zeta potential of −11.3 ± 1.4 mV, and an EE% of 75.7 ± 4.4 %. These results serve as a reference for comparing to data obtained from AP-CSLNs.

#### 3.2.4 *In vitro* release study

**Fig. 3:**
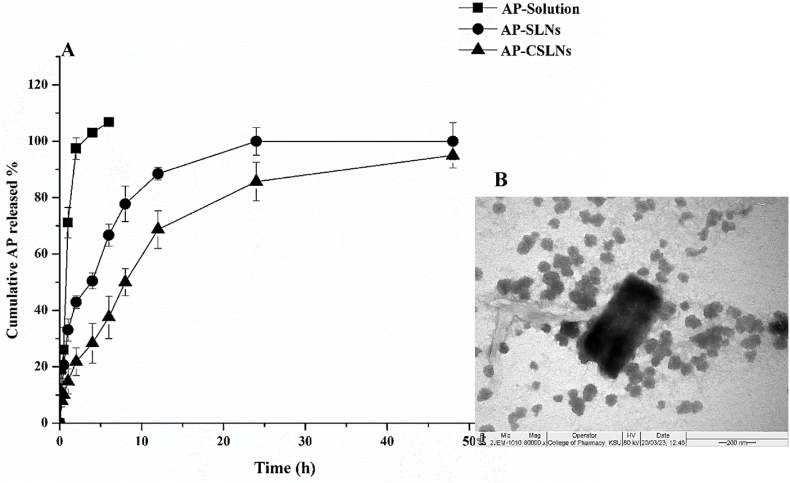
*In vitro* release pattern of AP-loaded SLNs and CSLNs in PBS (pH 7.4) with 0.2 % TW80 (A) and TEM images of the optimized AP-CSLNs formulation (B).

The dialysis bag technique is commonly used for *in vitro* drug release studies. The *in vitro* drug release profiles of AP-SLNs and CSLNs are shown in Fig. 3A. The free AP (control) showed as a complete released profile up to 4 h. On the other hand, the release profiles of AP-SLNs and AP-CSLNs displayed biphasic behavior, marked by an initial rapid release phase up to 4 h, followed by a slow release phase extending up to 48 h. Moreover, 66.7 % and 37.6 % of AP were released from AP-SLNs and AP-CSLNs, respectively, after 6 h. After 12 h, the release amounts of AP were increased to 88.5 % and 68.7 % from AP-SLNs and AP-CSLNs, respectively. AP was entirely released from AP-SLNs after 24 h compared to 86 % from AP-CSLNs. Such differences in the release profile between AP-SLNs and AP-CSLNs could be attributed to the effect of the particle size on AP-SLNs. The reduction of particle size results in an increased surface, which facilitates the rate of drug diffusion, as described by Fick's first law [35]. The observed initial rapid release of the drug may be due to the presence of AP on the NPs surface [34]. However, as the polymer undergoes hydration and expansion, a sustained release of the drug has been observed [36]. Moreover, AP-CSLNs demonstrated reduced rapid and sustained release, attributable to the inhibitory impact of the CS coating layer [34], which was proposed as a reason for the release pattern observed in CSLNs [34]. The reduction could be CS-coated could influence the presence of AP on the surfaces of NPs during preparation [37]. Also, the CS layer could impact the distribution and accessibility of AP within the CSLNs, reducing the release of AP compared to the investigated formulations, which control the release of AP. The CS-coated SLNs inhibited the burst release of ciprofloxacin and prolonged its release [38]. The possible mechanisms of this sustained release profile may be attributed to the gradual degradation of the CS layer [5,38]. The *in vitro* release experiments may not fully support the in vivo release behavior. Therefore, further in vivo studies would be required to consider the actual drug release.

Furthermore, to investigate the release kinetic model of AP-SLNs and CSLNs, different mathematical models were examined (Table 5). The highest value of the correlation coefficient (R^2^) was considered to identify the release kinetic model of AP. Table 5 demonstrated that the release of AP-SLNs fits with the first-order model, suggesting AP release is dependent on its concentration. Moreover, the release model of AP-CSLNs fits with Korsmeyer-Peppas's equation. Notably, the values of the release exponent (n value) were found to be less than 0.474, indicating the diffusion with a Fickian transport pattern. AP extract needs to penetrate the CS layer before being released from the CSLNs. The thickness of the CS coating can regulate the diffusion of the AP extract. Thus, several factors contribute to AP release from CSLNs, such as the CS coating, particle size, and magnitude of zeta potential [5]. These factors can be optimized to attain the favorite drug release and improve the therapeutic efficacy of the encapsulated AP. Moreover, the CS-coated NPs could interact with cell membranes to prolong their residence and augment drug absorption. According to the recent studies conducted by Aldayel et al. (2023) [5] and Shawky et al. (2022) [14], the CS surface-coated SLNs could magnify the biological activity of herbal drugs. However, further studies are required to fully understand the mechanisms involved and optimize the formulation parameters for achieving desired release profiles and therapeutic efficacy.

**Table 5:** *In vitro* release kinetics models of SLNs and CSLNs. (n = 3, mean ± SD).

Correlation coefficient (R2)					
Formulations	Zero-Order	First-Order	Higuchi's Model		Korsmeyer-Peppas Model
	R^2^				n
AP-SLNs	0.777	0.977	0.905	0.927	0.378
AP-CSLNs	0.913	0.973	0.962	0.984	0.474

TEM images of the optimized AP-CSLNs are presented in Fig. 3 B, which showed semispherical particles with uniform monodispersing with a rough surface. The absence of AP particles in the images might confirm a successful loading of AP inside NPs. No correlation was observed between the size of the particles present in the TEM image and that measured by dynamic light scattering.

### 3.2 Biological activities of AP-CSLNs and AP-SLNs

#### 3.2.1 Antioxidant activity

The optimized CSLNs were investigated for their potential to enhance the antioxidant activity of AP. The antioxidant activity of AP-CSLNs was evaluated through the DPPH assay, a wide technique used for measuring the effect of free radical scavenging [41].

**Fig. 4:**
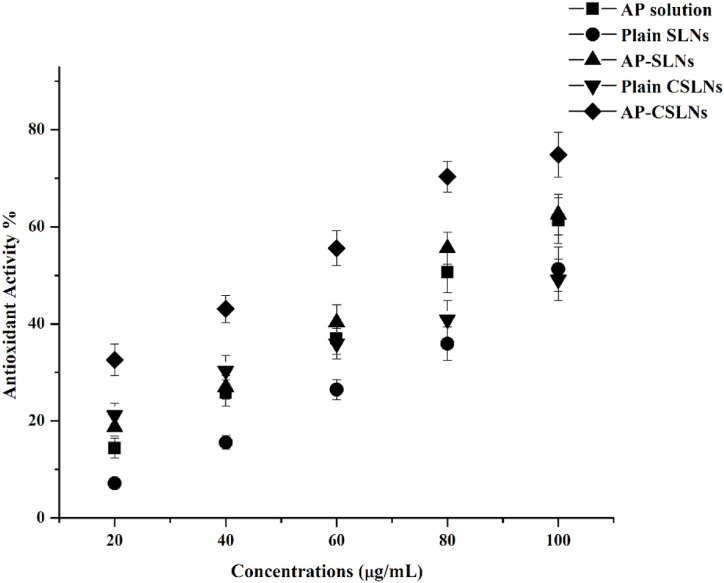
Antioxidant efficacy of different concentrations of AP-CSLNs, AP-SLNs, and AP solution.

Fig. 4 represents the antioxidant activity of the AP-CSLNs, AP-SLNS, plain NPs, and the pure AP solution. Overall, these results revealed that AP-CSLNs had a significant impact on the antioxidant activity of AP extract. AP-CSLNs demonstrated the highest antioxidant activity with a DPPH scavenging percentage of 74.9 % at 100 μg/mL, followed by AP-SLNs with a scavenging percentage of 62.9 % at the same concentration. The AP extract exhibited significant antioxidant activity of 62.1 % at a concentration of 100 μg/mL. The antioxidant activity of plain CSLNs and SLNs showed a DPPH scavenging percentage of 49.9 % and 51.8 % at 100 μg/mL, respectively. The IC50 values, which represent the concentration required to scavenge 50 % of DPPH radicals, were 53 μg/mL for AP-CSLNs, 75.3 μg/mL for AP-SLNs, 97.5 μg/mL for plain CSLNs, and 102.8 μg/mL for plain SLNs. In comparison, the IC50 value of the AP extract alone was 86.6 μg/mL. The results demonstrated a positive correlation between the concentration of AP and its antioxidant activity. As the concentration of the AP increases, the antioxidant activity of the AP- CSLNs increases with a remarkable DPPH scavenging activity.

The enhancement of the antioxidant activity of AP-CSLNs originated from the combination of two effects: the inherent antioxidant properties of AP itself and the enhanced delivery provided by the nanocarriers [39]. Sharma et al. (2019) detected higher antioxidant activity in curcumin-loaded NPs compared to free drugs [40]. The authors attributed this effect to the improvement in curcumin delivery and bioavailability, which were facilitated by NPs. In another study, the antioxidant activity of resveratrol-loaded NPs was much better than free resveratrol [41].

The positive impact of NPs on the antioxidant activity of molecules could be ascribed to the capability of NPs to protect the encapsulated drugs from degradation in harsh conditions and allow them to reach systemic circulation intact. Moreover, NPs can accumulate in specific target tissues, providing more antioxidants at the site of oxidative stress [40]. The positive charge on the surface of NPs could interact with the negative charge of the cell membrane, facilitating their cellular uptake and thereby improving their antioxidant performance [30]. Furthermore, AP extract is rich in bioactive compounds such as phenolic compounds, flavonoids, and carotenoids, which possess strong antioxidant capabilities [5]. These compounds have the ability to scavenge free radicals, inhibit oxidative stress, and protect against cellular damage caused by reactive oxygen species (ROS).

#### 3.2.2 Antimicrobial activity

**Fig. 5:**
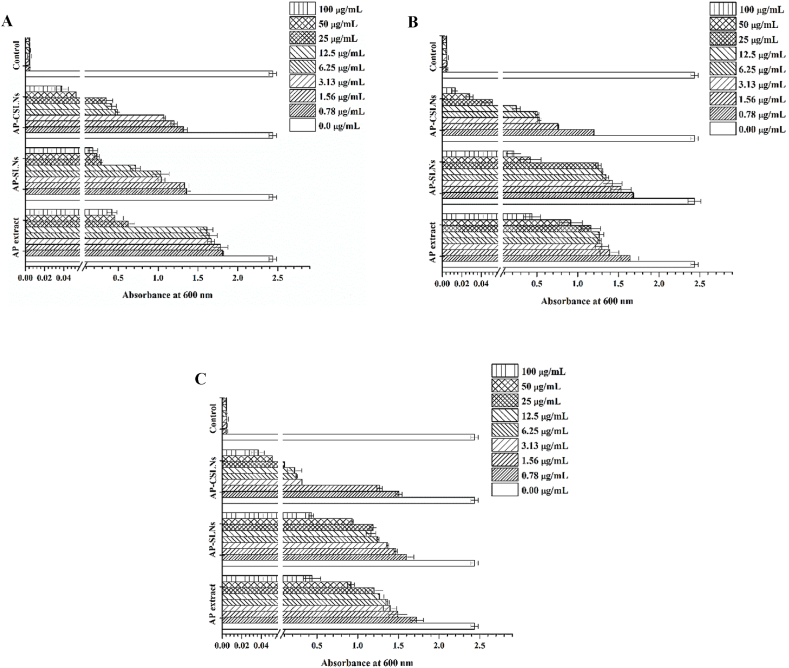
Antibacterial activity of AP extract, AP-SLNPs, AP-CSLNs, and positive control against (A) *S. aureus*, (B) *P. aeruginosa*, and (C) *E. coli*.

**Fig. 6:**
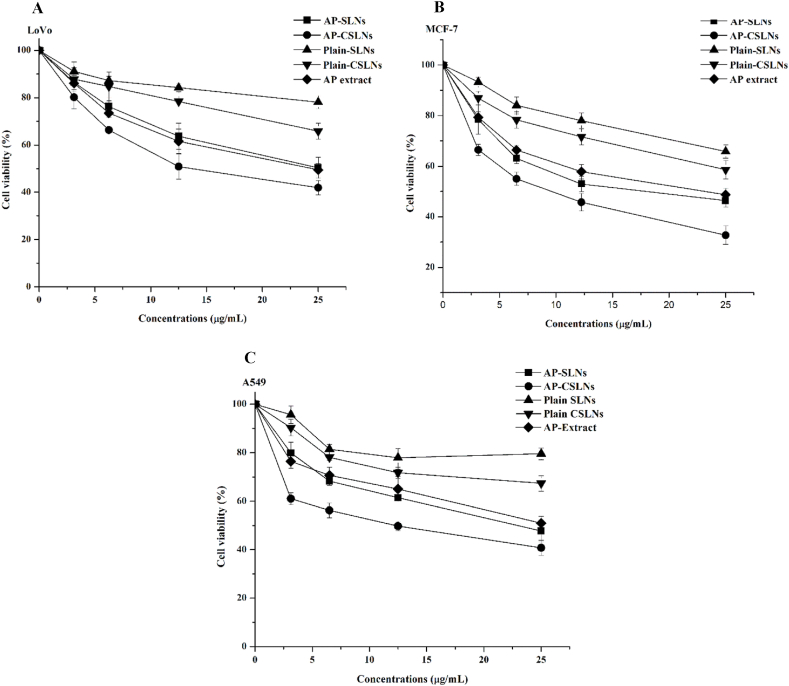
Cytotoxicity analysis of different cell lines(A) LoVo, (B) MCF-7, and A549 (C) treated with AP-SLN and AP-CSLNs at various concentrations for 48 h.

Plant extracts often contain essential compounds that have antimicrobial properties [4]. Moreover, the encapsulation of these extracts into nanocarriers such as SLNs could improve their antimicrobial activity. The combination of extracts, SLNs, and CS coating presents numerous advantages that contribute to their efficacy against bacteria [14]. It has been reported that CSLNs loaded with extracts exhibit promising potential for antimicrobial applications, attributed to their improved interaction with the bacterial cell membrane [5,8]. The microdilution broth assay was employed to evaluate the antimicrobial activity of AP extract, AP-SLNs, and AP-CSLNs. The efficacy of these formulations was compared with that of blank NPs. Their effectiveness against *S. aureus*, *P. aeruginosa,* and *E. coli* was inspected and represented in Fig. 6A, B, and C, respectively. Additionally, at a concentration of 200 μg/mL, the unloaded NPs exhibited no antimicrobial activity, whereas the loaded NPs displayed significant antimicrobial efficacy. Fig. 5 represents the results of testing the antimicrobial activity of AP extract, AP-SLNs, and AP-CSLNs. The results indicated the highest susceptibility of the tested microbes to the AP-NPs compared to the AP extract. Moreover, the antimicrobial effect demonstrated by AP confirmed the successful extraction of AP. Moreover, AP-CSLNs showed pronounced activity, indicating their potential to combat infections associated with the tested strains. These findings imply that the addition of AP to CSLNs improved the antimicrobial activity compared to other investigated formulations (Fig. 5). Hence, the synergy between CS and AP is crucial in improving the antibacterial properties of AP-CSLNs. Notably, plain CSLNs showed a clear antimicrobial effect against the tested bacterial strains, which confirmed the intrinsic antimicrobial properties of CS [5,30].

Regarding MIC values, AP-SLNs exhibited MICs at 25 μg/mL, 50 μg/mL, and 50 μg/mL for *S. aureus*, *P. aeruginosa*, and *E. coli*, respectively. In the case of AP-CSLNs, the values of MIC were 6.25 μg/mL, 12.5 μg/mL, and 25 μg/mL for *S. aureus*, *P. aeruginosa*, and *E. coli*, respectively. These data showed that AP-CSLNs effectively combat the tested microbes in this study. Furthermore, it has been reported that the utilization of mint or achillea millefolium loaded CSLNs resulted in increased antimicrobial activity against strains [42,43]. Therefore, the combination of AP, CS, and SLNs offers a more effective approach to combating microbial infections [44].

The improved antimicrobial efficacy is due to the nanoscale of the obtained preparations, which permits faster interaction with the cell wall of the microbe [5]. The positively charged NPs could facilitate interactions with the cell membrane of the microbe. Thus, CSLNs have emerged as a promising strategy and potential alternative to traditional antibiotics in treating bacterial infections. Additionally, more studies are required to evaluate the safety, stability, and long-term effects of these nanoparticles in a clinical setting. The antibacterial activity was performed using certain microorganisms, so it was limited to a short-term investigation, and the long-term effects may change.

3.2.3. Anticancer activity Natural products have traditionally inspired the development of innovative drug therapies, providing alternative and complementary options to conventional medications [45]. Therefore, the continued exploration of the potential activity of natural compounds, including AP extract, in cancer treatment is essential [46]. The potential anti-proliferative effects of AP extract have been investigated and confirmed [4,7]. To maximize the anticancer activity of plant extracts like AP, efficient nanocarriers are desirable [4,8]. Furthermore, AP-loaded nanocarriers, particularly using CS to modify the outer surface for further facilitating the binding to cancer cells and promoting the high cellular uptake of AP [5].

Thus, the antiproliferation effects of AP-SLNs, optimized AP-CSLNs, plain NPs (negative controls), and doxorubicin (positive control) were examined using various cell lines like LoVo (Fig. 6A), MCF-7 (Fig. 6B) and A549 (Fig. 6C). The cancer cell viability was determined after incubation with several concentrations of the obtained formulations (0–25 μg/mL) of AP for 48 h at 37 °C. The results revealed a dose-response pattern, which indicated that higher concentrations resulted in a greater reduction in cancer cell survival. Moreover, the plain NPs displayed minimal anticancer effects on the cancer cells tested. In contrast, AP-CSLNs showed a maximal anticancer effect compared to AP-SLNs and AP, particularly at a concentration of 25 μg/mL.

The obtained results indicated that the plain formulations did not achieve the IC50 within the range of AP concentrations that were tested. The minimum detectable anticancer activity of AP-CSLNs was determined to be 17.7 ± 1.5 for the LoVo cell lines, 10.2 ± 1.05 for MCF-7 cell lines, and 14.1 ± 0.9 μg/mL for A549 cell lines. Moreover, the AP-SLNs exhibited IC50 values of 24.3 ± 3.2 for the LoVo cell lines, 19.3 ± 0.8 for MCF-7 cell lines, and 22.9 ± 2.6 μg/mL for A549 cell lines. On the other hand, the AP extract exhibited an IC50 value of 23.2 ± 1.7, 17.9 ± 1.5, and 25.8 ± 1.9 for the LoVo, MCF-7, and A549 cell lines, respectively. Furthermore, the IC50 values of the reference standard (doxorubicin) were 0.95 ± 0.04, 0.53 ± 0.01, and 1.79 ± 0.13 μg/mL for the LoVo, MCF-7, and A549 cell lines, respectively. The results confirmed the significant cytotoxic activity of AP-CSLNs against multiple cancer cell lines. The great cytotoxic effect of AP-CSLNs is attributed to their better interaction with cancerous cells [5]. This interaction is primarily due to the presence of CS, which facilitates the delivery of AP, magnifying its cellular uptake and anticancer activity [13]. The anticancer activity was done using specific cell lines; the data observed may not be directly applicable to other cell types. However, further improvement and optimization are required to maximize the benefits of AP-CSLNs and address any potential risks.

## 4 Conclusions

The qualified AP-CSLNs were affected by the examined independent parameters of C-888, PL 188, and CS concentrations. The prepared CSLNs with a high content of C-888 and PL 188 significantly affected the particle size and EE% of AP, respectively. The utilization of CSLNs has emerged as a remarkably effective strategy for amplifying the biological activity of AP extract. In this study, SLNs and CSLNs can efficiently encapsulate AP extract and exhibit sustained release properties successfully. The inclusion of AP extract-CSLNs and SLNs led to a noteworthy improvement in their antioxidant activities. The loading of AP extract into CSLNs and SLNs resulted in a significant influence on its antioxidant activities. AP-CSLNs exhibited a synergistic enhancement in the antibacterial activity of tested bacteria (*S. aureus, P. aeruginosa,* and *E. coli*). Remarkably, AP-CSLNs demonstrated noteworthy antitumor activity against various cell lines like A549, LoVo, and MCF-7. The outcomes from this study reveal the possible use of AP-CSLNs as a hopeful carrier for AP due to their great antioxidant, antimicrobial, and antitumor activities. Future studies are required to explore the optimized nanoparticles' in vivo efficacy, safety, and therapeutic effect. Another exciting future research may be efficient to understand the mechanisms of the biological activities of these nanoparticles.

## Funding

This research project was funded by the Deanship of Scientific Research, 10.13039/501100004242Princess Nourah bint Abdulrahman University, through the Funding program for targeted research number (PNU-Targeted-4).

## Data availability statement

Data included in article/supp. material/referenced in article

## CRediT authorship contribution statement

**Tahany Saleh Aldayel:** Conceptualization, Resources, Writing - original draft, Funding acquisition. **Mohamed M. Badran:** Data curation, Methodology, Visualization, Writing - original draft. **Abdullah H. Alomrani:** Methodology, Writing - original draft. **Nora A. AlFaris:** Writing - original draft. **Jozaa Z. Altamimi:** Investigation, Methodology. **Ali S. Alqahtani:** Investigation, Methodology. **Fahd A. Nasr:** Investigation, Methodology. **Safina Ghaffar:** Investigation, Methodology. **Raha Orfali:** Investigation, Methodology, Writing - original draft.

## Declaration of competing interest

The authors declare the following financial interests/personal relationships which may be considered as potential competing interests:Tahany Saleh Aldayel reports financial support was provided by 10.13039/501100004242Princess Nourah bint Abdulrahman University. The authors declare no conflict of interest.
